# A Comparative Observational Study of the Diagnostic Utility of Impulse Oscillometry Versus Spirometry in Obstructive Airway Diseases

**DOI:** 10.7759/cureus.70589

**Published:** 2024-10-01

**Authors:** Souvik Sarkar, Ulhas Jadhav, Babaji Ghewade, Syamal Sarkar

**Affiliations:** 1 Respiratory Medicine, Jawaharlal Nehru Medical College, Datta Meghe Institute of Higher Education and Research, Wardha, IND

**Keywords:** asthma, chronic obstructive pulmonary disease (copd), impulse oscillometry (ios), lung function assessment, obstructive airway diseases, spirometry

## Abstract

Background

Obstructive airway diseases, including asthma and chronic obstructive pulmonary disease (COPD), significantly impact respiratory function, making accurate diagnosis and differentiation essential for proper management. While spirometry is the gold standard for assessing lung function, impulse oscillometry (IOS) has emerged as a complementary tool, especially when spirometry results are inconclusive. This study aimed to compare the diagnostic utility of IOS with spirometry in patients with obstructive airway diseases and evaluate the correlation between these two methods.

Methods

A comparative observational study was conducted over 18 months at a tertiary care hospital in central India, including 130 patients (65 with asthma and 65 with COPD). Diagnostic evaluations using spirometry and IOS were performed before and after bronchodilator administration. Spirometry parameters assessed were forced expiratory volume in one second (FEV1), forced vital capacity (FVC), and FEV1/FVC ratio, while IOS parameters evaluated included resistance at 5 Hz (R5), resistance at 20 Hz (R20), resonant frequency (Fres), reactance at 5 Hz (X5), and the area under the reactance curve (AX). Statistical analysis was conducted using IBM SPSS version 27.0 (IBM Corp., Armonk, USA) and GraphPad Prism version 7.0 (Dotmatics, Boston, USA).

Results

Significant differences were observed in spirometry parameters between asthma and COPD groups, with asthma patients showing better lung function (FEV1, FVC, and FEV1/FVC; p<0.05). No significant differences were found in IOS parameters between the groups except for a correlation between FEV1 (%) and IOS measurements in the asthma group. Spirometry demonstrated superior sensitivity in identifying airway obstruction compared to IOS. However, IOS was more effective in detecting peripheral airway obstruction in asthma patients, with 22 out of 65 (33.85%) asthma patients showing peripheral airway obstruction compared to six out of 65 (9.23%) COPD patients (p=0.001).

Conclusion

While spirometry remains the primary diagnostic tool for assessing obstructive airway diseases, IOS is a valuable adjunct, particularly for detecting peripheral airway involvement in asthma patients. Combining spirometry and IOS enhances diagnostic accuracy and provides a more comprehensive assessment of lung function in patients with asthma and COPD.

## Introduction

Obstructive airway diseases, such as asthma and chronic obstructive pulmonary disease (COPD), are among the most common chronic respiratory conditions globally, imposing a substantial burden on healthcare systems and affecting millions of individuals. Asthma is characterized by variable and reversible airway obstruction, airway hyperresponsiveness, and inflammation [1]. COPD, on the other hand, is a progressive and largely irreversible condition marked by persistent airflow limitation and is often associated with a history of exposure to noxious particles or gases, particularly from tobacco smoke [2]. According to the Global Burden of Disease Study, COPD was the third leading cause of death worldwide in 2019, and asthma continues to affect over 300 million people globally [3,4]. Both conditions contribute significantly to morbidity, mortality, and reduced quality of life for patients.

Accurate diagnosis is paramount in distinguishing between asthma and COPD, as the management and prognosis of these conditions differ considerably. Traditionally, spirometry has been the cornerstone diagnostic test for obstructive airway diseases. Spirometry measures parameters such as forced expiratory volume in one second (FEV1), forced vital capacity (FVC), and FEV1/FVC ratio, which are crucial for assessing the degree of airflow obstruction [5]. The reversibility of airflow obstruction, as assessed by bronchodilator testing, is often used to differentiate asthma from COPD [6]. However, spirometry has limitations, particularly in patients who cannot perform the forced manoeuvres required for accurate readings, such as young children, elderly individuals, or those with comorbid conditions [7]. Moreover, spirometry primarily measures central airway function and may not be sensitive enough to detect early-stage small airway dysfunction, often present in asthma and COPD [8].

In recent years, impulse oscillometry (IOS) has gained recognition as a complementary or alternative tool for assessing lung function. IOS is a non-invasive method that measures respiratory impedance by applying pressure oscillations during normal tidal breathing [9]. This technique provides information on airway resistance (R) and reactance (X) at different frequencies, offering insights into both central and peripheral airway function [10]. Unlike spirometry, IOS requires minimal patient cooperation, making it particularly useful for populations that struggle with forced respiratory manoeuvres [11]. Additionally, IOS has shown potential in identifying early small airway dysfunction, a key feature of both asthma and COPD that may be missed by spirometry [12].

Several studies have demonstrated that IOS can be a valuable tool in diagnosing obstructive airway diseases, particularly when spirometry results are inconclusive [13,14]. IOS has been shown to correlate well with spirometric indices in patients with asthma and COPD, and it can provide additional diagnostic information about peripheral airway involvement [15]. For instance, studies have highlighted that IOS may be more sensitive in detecting peripheral airway dysfunction in asthmatic patients, while spirometry is more effective for identifying central airway obstruction [16]. Furthermore, IOS offers a unique advantage in assessing bronchodilator response, as it measures airway resistance and reactance changes, which may offer a more comprehensive understanding of lung function [17].

Given the advantages of spirometry and IOS, comparing their diagnostic utility in a clinical setting is essential. This study assesses the comparative efficacy of IOS versus spirometry in patients with obstructive airway diseases, including asthma and COPD. This study seeks to provide a more comprehensive approach to diagnosing and managing these chronic respiratory conditions by evaluating the correlations between spirometry and IOS parameters. Ultimately, this could lead to better treatment strategies and patient outcomes, especially in populations with challenging spirometry.

## Materials and methods

Study setting

The study was conducted in a tertiary care hospital, Acharya Vinoba Bhave Rural Hospital (AVBRH), affiliated with Jawaharlal Nehru Medical College, Sawangi Meghe, located in the central region of India. The hospital serves as a referral center for patients from urban and rural regions, providing a comprehensive setting for evaluating patients with obstructive airway diseases such as asthma and COPD.

Study design, duration, and sample size

This study employed a comparative observational design and was conducted over 18 months, from August 2022 to March 2024. The study included patients attending the outpatient department (OPD) or admitted to the inpatient department (IPD) of the respiratory medicine department at AVBRH. Patients were selected based on the inclusion and exclusion criteria outlined below. The sample size for this study was calculated using Daniel’s formula, based on a COPD prevalence of 6.5% and a desired margin of error of 6%. As a result, 65 patients were required in each group, resulting in 130 participants (65 with asthma and 65 with COPD).

Inclusion and exclusion criteria

The study included patients who were clinically suspected of having obstructive airway diseases, specifically asthma and COPD. To be eligible for inclusion, patients had to be at least six years old and capable of understanding and performing the required diagnostic investigations. This ensured that all participants could actively and accurately participate in the study procedures, such as spirometry and IOS, without difficulties. Patients were excluded from the study if they were positive for tuberculosis, as this condition could complicate the assessment of obstructive airway diseases. Additionally, those who had recently undergone abdominal, thoracic, eye, or ear surgeries were excluded to avoid potential complications that could interfere with pulmonary function testing. Individuals with orofacial abnormalities that might prevent the proper execution of spirometry or IOS were not included. Furthermore, due to the ongoing global pandemic, patients suspected of having COVID-19 or those with a positive reverse transcription-polymerase chain reaction (RT-PCR) test were excluded to prevent contamination risks and ensure the safety of the participants and healthcare staff. Lastly, any patient unwilling to provide informed consent was not included in the study by ethical standards.

Study population, sampling, and diagnostic methods

A total of 130 patients were included in the study, comprising 65 asthmatic and 65 COPD patients. The diagnosis of asthma or COPD was based on patient history, physical examination findings, and chest X-ray results. The diagnosis was further supported by the guidelines from the Global Initiative for Asthma (GINA) [18] and the Global Initiative for Chronic Obstructive Lung Disease (GOLD) [19]. All participants were informed about the study objectives, and written consent was obtained from each patient before their inclusion. All patients underwent detailed clinical evaluations, including medical history, physical examination, and chest X-ray. Diagnostic procedures involved the use of spirometry and IOS [20] to assess lung function. The following parameters were evaluated: spirometry was conducted using the RMS Helios-401 handheld turbine mechanism machine (RMS, Haryana, India) [21]. Parameters such as FEV1, FVC, and the FEV1/FVC ratio were recorded. The procedure followed the American Thoracic Society/European Respiratory Society (ATS/ERS) Task Force 2005 guidelines [22], ensuring quality control through acceptability and repeatability criteria. Spirometry was performed before and after administering bronchodilator (200 mcg of levosabutamol) to assess bronchodilator reversibility. IOS was conducted using the Master Screen-IOS (Erich Jaeger, Germany) [23]. The parameters evaluated included resistance at 5 Hz (R5), resistance at 20 Hz (R20), the difference between R5 and R20 (R5-R20), resonant frequency (Fres), reactance at 5 Hz (X5), and the area under the reactance curve (AX). Each patient performed the test in a seated position, holding the mouthpiece with a tight seal to avoid air leakage, while their cheeks were supported by their hands to prevent upper airway impedance. IOS measurements were taken before and after administering the bronchodilator (levosabutamol), similar to the spirometry procedure.

Diagnostic criteria and statistical analysis

The diagnosis of COPD was based on a history of progressive exertional or persistent dyspnea, chronic cough (intermittent or non-productive), chronic sputum production, and exposure to risk factors such as tobacco smoke or occupational hazards. The diagnosis of asthma was based on a history of recurrent symptoms, such as wheezing, chest tightness, or difficulty breathing, often triggered by common allergens or exercise. All diagnostic criteria adhered to the GOLD and GINA guidelines. Descriptive and inferential statistics were employed for data analysis using IBM SPSS version 27.0 (IBM Corp., Armonk, USA) and GraphPad Prism version 7.0 (Dotmatics, Boston, USA). The Chi-square test was used for categorical variables, and Student’s t-test was applied to compare continuous variables between the two groups. Data were expressed as mean ± standard deviation (SD), and correlations between IOS and spirometry measurements were assessed using Pearson’s correlation coefficient for normally distributed data and Spearman’s rank correlation for non-normally distributed data. A p-value of <0.05 was considered statistically significant.

Ethical clearance

Ethical approval for the study was obtained from the ethical committee of Datta Meghe Institute of Medical Sciences (Deemed University), Sawangi Meghe, Wardha. The study protocol and design were reviewed and approved with the corresponding approval letter numbered DMIMS(DU)/IEC/2022/74. The study's methodology and relevance were explained to all patients who met the selection criteria. Written informed consent was obtained from all eligible study subjects before they participated in the study.

## Results

Table 1 presents the age distribution of the study population. A significant proportion of COPD patients, 61 out of 65 (93.85%), were aged between 41 and 80 years, whereas asthma patients were more evenly distributed across a younger age range, with 26 out of 65 (40%) in the 17-40 years range. This significant difference in age distribution between the groups was statistically significant (p=0.0001).

**Table 1:** Distribution of patients in the asthma and COPD groups according to their age in years COPD: chronic obstructive pulmonary disease

Age Group	Asthma	COPD	ꭓ^2^ value	p value
6-16 yrs	5 (7.69%)	0 (0%)	34.24	0.0001, S
17-40 yrs	26 (40%)	2 (3.08%)		
41-80 yrs	33 (50.77%)	61 (93.85%)		
>80 yrs	1 (1.54%)	2 (3.08%)		
Total	65 (100%)	65 (100%)		
Mean±SD	44.09±18.60	61.38±12.51		
Range	6-83 yrs	25-86 yrs		

Table 2 shows the gender distribution of the study population. Males were predominant in both groups, with 43 out of 65 (66.15%) COPD patients and 37 out of 65 (56.92%) asthma patients being male. However, the difference in gender distribution between the two groups was not statistically significant (p=0.27).

**Table 2:** Gender-wise distribution of patients in the the asthma and COPD groups COPD: chronic obstructive pulmonary disease

Gender	Asthma	COPD	ꭓ^2 ^value	p value
Male	37 (56.92%)	43 (66.15%)	1.17	0.27, NS
Female	28 (43.08%)	22 (33.85%)		
Total	65 (100%)	65 (100%)		

Table 3 shows the clinical presentation of the study population. All patients in both groups experienced breathlessness. Wheezing was observed exclusively in asthma patients, with eight out of 65 (12.31%) showing this symptom, while none of the COPD patients had wheezing. Cough and expectoration were similarly distributed between the two groups, with no significant difference. However, the occurrence of breathlessness was significantly different between the groups (p=0.013).

**Table 3:** Distribution of patients in the asthma and COPD groups according to clinical presentation COPD: chronic obstructive pulmonary disease

Clinical Presentation	Asthma	COPD	ꭓ^2^ value	p value
Breathlessness	65 (100%)	65 (100%)	10.73	0.013, S
Cough	12 (18.46%)	11 (16.92%)		
Wheezing	8 (12.31%)	0 (0%)		
Expectoration	3 (4.62%)	2 (3.08%)		

Table 4 shows risk factors for smoking and past tuberculosis were significantly higher in COPD patients, while allergies were more common in asthma patients (p=0.0001). Biomass exposure was also more frequent in COPD (p=0.024).

**Table 4:** Distribution of patients in the asthma and COPD groups according to risk factors COPD: chronic obstructive pulmonary disease; TB: tuberculosis; H/O: history of

Risk Factors	Asthma	COPD	ꭓ^2^ value	p value
Past H/O TB	3 (4.62%)	23 (35.38%)	19.23	0.0001, S
H/O Smoking	8 (12.31%)	30 (46.15%)	17.99	0.0001, S
H/O Allergies	60 (92.31%)	10 (15.38%)	77.38	0.0001, S
Biomass Exposure	15 (23.08%)	27 (41.54%)	5.06	0.024, S

Table 5 presents spirometry parameters. Asthma patients showed better spirometric parameters than COPD patients, including FVC, FEV1, and FEV1/FVC ratios (p=0.0001). This indicates more pronounced airflow limitation in COPD.

**Table 5:** Comparison of spirometry parameters in the asthma and COPD groups COPD: chronic obstructive pulmonary disease; FEV1: forced expiratory volume in one second; FVC: forced vital capacity; FEF_25-75_: forced mid-expiratory flow

Spirometry Parameters	Asthma	COPD	t value	p value
FVC (L)	2.06±0.88	1.31±0.50	5.87	0.0001, S
FVC (%)	92.53±19.76	73.30±13.26	6.51	0.0001, S
FEV1 (L)	1.48±0.73	0.85±0.36	6.26	0.0001, S
FEV1 (%)	66.53±22.09	45.67±14.47	6.36	0.0001, S
FEV1/FVC (%)	70.92±16.38	62.38±21.83	2.52	0.013, S
FEF_25-75_ (%)	40.70±22.66	28.08±21.08	3.21	0.001, S

Table 6 shows oscillometry parameters. No significant differences were observed between asthma and COPD patients in oscillometry measures (R5, R20, Fres, X5, AX), indicating similar airway resistance profiles.

**Table 6:** Comparison of oscillometry parameters in the asthma and COPD groups COPD: chronic obstructive pulmonary disease; R5: resistance at 5 Hz; R20: resistance at 20 Hz; R5-R20: difference between R5 and R20; Fres: resonant frequency; X5: reactance at 5 Hz; AX: area under the reactance curve

Oscillometry Parameters	Asthma	COPD	t value	p value
R5 (kpa.s/l)	0.93±0.78	0.93±0.86	0.01	0.99, NS
R20 (kpa.s/l)	0.54±0.37	0.53±0.38	0.12	0.90, NS
R5-R20 (kpa.s/l)	0.39±0.51	0.39±0.53	0.07	0.94, NS
Fres	18.67±13.29	22.01±13.01	1.44	0.15, NS
X5 (kpa.s/l)	-0.21±0.24	-0.19±0.59	0.29	0.76, NS
AX	2.50±5.17	2.16±2.27	0.48	0.62, NS

Table 7 shows oscillometry changes pre- and post-bronchodilator. There were no significant changes in oscillometry parameters in both groups after bronchodilator treatment, suggesting similar airway responsiveness between asthma and COPD (p>0.05).

**Table 7:** Comparison of mean percentage of change in oscillometry parameters in the asthma and COPD groups pre- and post-treatment with bronchodilator COPD: chronic obstructive pulmonary disease; R5: resistance at 5 Hz; R20: resistance at 20 Hz; R5-R20: difference between R5 and R20; Fres: resonant frequency; X5: reactance at 5 Hz; AX: area under the reactance curve

Oscillometry Parameters	Asthma	COPD	t value	p value
R5 (kpa.s/l)	-10.83±28.44	-7.72±30.69	0.59	0.55, NS
R20 (kpa.s/l)	-4.21±26.36	-5.92±26.81	0.36	0.71, NS
R5-R20 (kpa.s/l)	-8.70±110.25	-7.13±51.92	0.10	0.91, NS
Fres	21.89±134.08	-8.75±33.99	1.78	0.07, NS
X5 (kpa.s/l)	-276.89±777.16	-134.52±331.48	1.36	0.17, NS
AX	9.72±216.05	-35.63±266.96	1.06	0.28, NS

Table 8 shows the correlation of FEV1 (%) with oscillometry parameters in asthma; a significant negative correlation was observed between FEV1 and R5 and R20 (p<0.05). No significant correlations were found in COPD, indicating differing respiratory mechanics.

**Table 8:** Correlation of FEV1 (%) with oscillometry parameters in the asthma and COPD groups COPD: chronic obstructive pulmonary disease; R5: resistance at 5 Hz; R20: resistance at 20 Hz; R5-R20: difference between R5 and R20; Fres: resonant frequency; X5: reactance at 5 Hz; AX: area under the reactance curve

Oscillometry Parameters	Asthma		COPD	
	r value	p value	r value	p value
R5 (kpa.s/l)	-0.288	0.020, S	0.010	0.934, NS
R20 (kpa.s/l)	-0.304	0.014, S	0.016	0.900, NS
R5-R20 (kpa.s/l)	-0.224	0.073, NS	0.006	0.961, NS
Fres	-0.132	0.295, NS	0.008	0.947, NS
X5 (kpa.s/l)	0.121	0.338, NS	0.108	0.391, NS
AX	-0.233	0.062, NS	-0.125	0.320, NS

Table 9 shows the R5-R20 comparison with FEF_25-75_. Patients with R5-R20≥0.1 exhibited significant peripheral airway obstruction (p=0.0001), highlighting the relationship between oscillometry and spirometry.

**Table 9:** Comparison of R5-R20 (kpa.s/l) with FEF25-75 FEF_25-75_: forced mid-expiratory flow; R5-R20: difference between resistance at 5 Hz (R5) and resistance at 20 Hz (R20)

R5-R20 (kpa.s/l)	FEF_25-75_			ꭓ^2^ value	p value
	<65%	≥65%	Total		
≥0.1	32 (96.96%)	0 (0%)	32 (96.96%)	33	0.0001, S
<0.1	0 (0%)	1 (3.04%)	1 (3.04%)		
Total	32 (96.96%)	1 (3.04%)	33 (100%)		

Table 10 shows the distribution of peripheral airway obstruction among patients based on oscillometry results, i.e., R5-R20>0.1. A total of 28 patients exhibited peripheral airway obstruction. Of these, the majority had early small airway disease, with eight out of 28 (28.57%) patients diagnosed. In comparison, nine out of 28 (32.14%) were classified as having obstructive airway disease with poor bronchodilator response. Four patients had good bronchodilator response and seven patients had normal spirometric values.

**Table 10:** Distribution of patients having peripheral airway obstruction on oscillometry with their spirometric diagnosis OAD: obstructive airway disease; IOS: impulse oscillometry; BDR: bronchodilator response

Spirometric Diagnosis	Number of Patients Having Peripheral Airway Obstruction on IOS	Percentage
Early small airway disease	8	28.57
OAD with good BDR	4	14.29
OAD with poor BDR	9	32.14
Normal	7	25
Total	28	100

Table 11 illustrates the diagnosis based on oscillometry findings. Large airway obstruction was more prevalent among COPD patients, with 10 out of 65 (15.38%) showing this pattern. In contrast, asthma patients exhibited a higher incidence of peripheral airway obstruction, observed in 22 out of 65 (33.85%). This significant difference in airway involvement patterns between asthma and COPD patients was statistically significant (p=0.001). Total airway obstruction on IOS was equally distributed in both asthma (34) and COPD (39) patients.

**Table 11:** Comparison between final diagnosis by oscillometry in asthma and COPD patients COPD: chronic obstructive pulmonary disease

Final Diagnosis	Asthma	COPD	ꭓ^2^ value	p value
Large airway obstruction	1 (1.54%)	10 (15.38%)	17.07	0.001, S
Peripheral airway obstruction	22 (33.85%)	6 (9.23%)		
Total airway obstruction	34 (52.31%)	39 (60%)		
Normal	8 (12.31%)	10 (15.38%)		
Total	65 (100%)	65 (100%)		

## Discussion

This study aimed to compare the diagnostic utility of IOS and spirometry in assessing lung function in patients with obstructive airway diseases, including asthma and COPD. Our findings reinforce the established role of spirometry as the gold standard in diagnosing these conditions while highlighting IOS as a valuable complementary tool, particularly for detecting peripheral airway obstruction, which spirometry may overlook. Spirometry remains the cornerstone of pulmonary function testing due to its ability to measure key lung function parameters such as FEV1, FVC, and the FEV1/FVC ratio, which are crucial in diagnosing and monitoring obstructive airway diseases. Our study observed significant differences in spirometry results between the asthma and COPD groups, particularly regarding FEV1 and FEV1/FVC ratios. Patients with asthma had better lung function, as indicated by higher mean values for FEV1, FVC, and FEV1/FVC ratio compared to COPD patients (p<0.05). This is consistent with the literature, which often shows that asthma, due to its episodic and reversible nature, tends to have less permanent damage to lung function compared to COPD, where chronic, progressive airflow limitation is common [1,2].

However, spirometry does have its limitations, particularly in detecting small or peripheral airway obstruction. This limitation is critical because small airway disease is often the earliest manifestation of obstructive lung disease and can be present even when spirometric values are within the normal range [24,11]. In our study, seven patients who were clinically diagnosed with obstructive airway disease had normal spirometry, but IOS demonstrated early small airway disease. Our study demonstrated that IOS, which measures respiratory system impedance, is particularly useful in detecting peripheral airway obstruction. About 33.85% of asthma patients in our study showed evidence of peripheral airway obstruction on IOS compared to only 9.23% of COPD patients (p=0.001). This finding aligns with earlier studies, which suggested that IOS is more sensitive than spirometry for detecting early or small airway disease, particularly asthma [13,25]. IOS offers several practical advantages over spirometry. Unlike spirometry, IOS does not require forceful exhalation, making it a more patient-friendly test, particularly for individuals who are very young, elderly, or have severe respiratory impairment [26]. In our study, IOS was successfully performed in all participants, including those with more advanced diseases, where spirometry could be challenging. This advantage is particularly relevant for patients with severe COPD, where hyperinflation and air trapping can make it difficult to generate the forced expiratory maneuvers required for spirometry [12].

Another key finding of our study is the significant correlation between FEV1 (%) and certain IOS parameters, particularly in the asthma group. R5 and R20, which represent resistance at low and high frequencies, respectively, were inversely correlated with FEV1 (%), suggesting that higher resistance values on IOS are associated with worse lung function as measured by spirometry. This relationship was particularly pronounced in the asthma group, consistent with previous studies showing that IOS can detect changes in airway resistance corresponding to changes in spirometric parameters [9,27]. However, this correlation was insignificant in the COPD group, indicating that IOS may be less sensitive for detecting central airway obstruction in COPD. This could be because COPD, especially in its advanced stages, is characterized by more widespread and heterogeneous airway obstruction, which IOS may not as easily capture [28]. While IOS has demonstrated clear benefits, it is important to recognize its limitations. The IOS parameters, such as R5 and R20, did not show significant differences between asthma and COPD groups in our study except for the correlation with FEV1 (%) in the asthma group. This lack of differentiation suggests that IOS, while useful in detecting small airway disease, may not be as effective in distinguishing between different types of obstructive airway diseases. For example, large airway obstructions, which are more common in COPD, may not be as easily detected by IOS [29]. Thus, while IOS provides valuable information about peripheral airway function, it should be considered an adjunct rather than a replacement for spirometry.

The clinical implications of these findings are significant. For asthma patients, particularly those with early or mild disease, IOS can provide crucial information about small airway involvement that spirometry might miss. By identifying peripheral airway obstruction early, clinicians can adjust treatment strategies, potentially preventing disease progression [30]. In COPD patients, while spirometry remains the primary tool for assessing central airway obstruction, IOS can be useful in cases where spirometry results are inconclusive or difficult to obtain. The combination of spirometry and IOS offers a more comprehensive assessment of central and peripheral airway function, leading to more personalized and effective treatment plans [31].

Study strengths and limitations

The strengths of this study lie in its comparative approach, which provides valuable insights into the respective roles of spirometry and IOS in diagnosing and managing obstructive airway diseases. By including both asthma and COPD patients, we were able to highlight the unique advantages of each test in different disease contexts. Additionally, the use of bronchodilator reversibility testing in both spirometry and IOS adds robustness to our findings as it allows for the assessment of airway responsiveness, which is a key feature in distinguishing between asthma and COPD [5]. However, several limitations must be acknowledged. First, the sample size, while calculated to be statistically adequate, may still limit the generalizability of our results. A larger cohort might have provided more definitive conclusions, particularly regarding the role of IOS in COPD. Second, the study was conducted in a single tertiary care hospital, which may not reflect the diversity of patients seen in other clinical settings. Third, we excluded patients with conditions such as tuberculosis and recent surgeries, which could have impacted the applicability of IOS in more complex cases. Finally, the study did not include longitudinal follow-up, which could have provided valuable data on how IOS and spirometry predict disease progression and treatment outcomes over time.

## Conclusions

This study underscores the complementary roles of IOS and spirometry in diagnosing and assessing obstructive airway diseases, particularly asthma and COPD. While spirometry remains the gold standard for measuring central airway obstruction and bronchodilator response, IOS offers distinct advantages in assessing peripheral airway function and is particularly useful for patients who struggle to perform spirometry, such as young children, the elderly, and those with severe respiratory impairment. The findings suggest that IOS can detect subtle changes in airway resistance and reactance, making it a valuable adjunct in identifying early small airway involvement that spirometry may not capture. Combining both diagnostic tools provides a more comprehensive evaluation of lung function, enhancing the accuracy of differentiation between asthma and COPD. This research is important because it highlights the potential for integrating IOS into routine clinical practice alongside spirometry, which could significantly improve diagnostic accuracy and patient management. By identifying small airway disease earlier, clinicians can intervene sooner, potentially altering the course of these chronic conditions and improving patient outcomes. Further research is necessary to explore the full potential of IOS across diverse clinical settings and to solidify its role in routine respiratory disease evaluation.
